# A Computer-Assisted Diagnostic Method for Accurate Detection of Early Nondisplaced Fractures of the Femoral Neck

**DOI:** 10.3390/biomedicines11113100

**Published:** 2023-11-20

**Authors:** S. L. Hsieh, J. L. Chiang, C. H. Chuang, Y. Y. Chen, C. J. Hsu

**Affiliations:** 1 Minimally Invasive Spine and Joint Center, Buddhist Tzu Chi General Hospital Taichung Branch, Taichung 427213, Taiwan; miscyy@seed.net.tw; 2 College of Electrical Engineering and Computer Science, National Chin-Yi University of Technology, Taichung 411030, Taiwan; cjunglung@ncut.edu.tw (J.L.C.); chchuang@ncut.edu.tw (C.H.C.); 3 Department of Orthopedic Surgery, China Medical University Hospital, Taichung 404327, Taiwan; d5983@mail.cmuh.org.tw

**Keywords:** radiograph, nondisplaced, femoral neck fracture, deep learning network

## Abstract

Nondisplaced femoral neck fractures are sometimes misdiagnosed by radiographs, which may deteriorate into displaced fractures. However, few efficient artificial intelligent methods have been reported. We developed an automatic detection method using deep learning networks to pinpoint femoral neck fractures on radiographs to assist physicians in making an accurate diagnosis in the first place. Our proposed accurate automatic detection method, called the direction-aware fracture-detection network (DAFDNet), consists of two steps, namely region-of-interest (ROI) segmentation and fracture detection. The first step removes the noise region and pinpoints the femoral neck region. The fracture-detection step uses a direction-aware deep learning algorithm to mark the exact femoral neck fracture location in the region detected in the first step. A total of 3840 femoral neck parts in anterior–posterior (AP) pelvis radiographs collected from the China Medical University Hospital database were used to test our method. The simulation results showed that DAFDNet outperformed the U-Net and DenseNet methods in terms of the IOU value, Dice value, and Jaccard value. Our proposed DAFDNet demonstrated over 94.8% accuracy in differentiating non-displaced Garden type I and type II femoral neck fracture cases. Our DAFDNet method outperformed the diagnostic accuracy of general practitioners and orthopedic surgeons in accurately locating Garden type I and type II fracture locations. This study can determine the feasibility of applying artificial intelligence in a clinical setting and how the use of deep learning networks assists physicians in improving correct diagnoses compared to the current traditional orthopedic manual assessments.

## 1. Introduction

**Figure 1:**
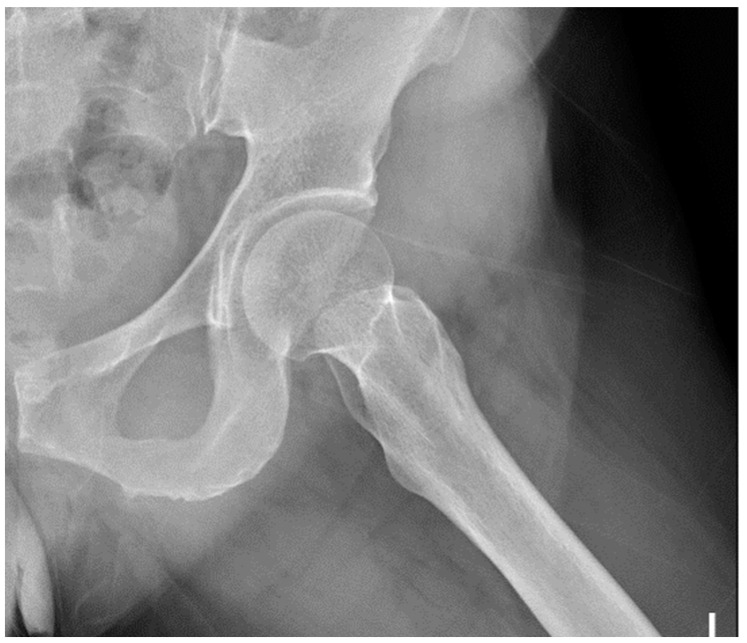
A normal lateral view of the pelvis and proximal femur.

A femoral neck fracture (FNF) is one of the most common osteoporotic fractures in the elderly, and it causes substantial morbidity and mortality [1,2,3]. Figure 1 shows a normal lateral view of the pelvis and proximal femur. According to the radiograph-based Garden classification system for assessing fracture severity, FNFs can be classified into four types, namely nondisplaced Garden I and II and displaced Garden III and IV [4]. The Garden classification incorporates the displacement, fracture completeness, and relationship of bony trabeculae in the femoral head and neck. Type I is described as a nondisplaced fracture with a valgus-impacted incomplete fracture and a disruption in the lateral cortex, while the medial cortex is preserved. Type II is described as a complete fracture without displacement. Type III is described as a complete fracture with partial displacement, indicated by a change in the angle of the trabeculae. Type IV is described as a complete fracture with complete displacement. The features of displaced FNFs are clinically distinct and distinct through imaging, whereas those of nondisplaced FNFs are challenging and receive less attention [5,6,7,8]. The radiographic imaging of nondisplaced FNFs can be compromised by osteoporosis, obesity, patient position-related reasons, the use of portable radiographic equipment, and poor image quality, which creates additional difficulties for clinicians [6,9].

In order to understand the misdiagnosis rate of nondisplaced FNFs in radiographs, we conducted a trial at the China Medical University Hospital (CMUH). One ER doctor, one junior PGY-1 doctor, and one ten-years-of-experience senior orthopedic doctor volunteered to read 480 pelvis AP-view X-rays with nondisplaced FNFs. The diagnosis of fractures was based on a further pelvis CT scan and radiologist reports. The overall misrecognized rates of nondisplaced FNFs were 7.87% for the PGY doctor, 4.19% for the ER doctor, and 2.44% for the senior orthopedic doctor. Table 1 shows the results compared with previous reviews. The misdiagnosis rate was higher for the junior doctor and ER doctor compared with the senior orthopedic doctor. Therefore, AI-assisted diagnoses of radiographs are helpful for alerting doctors in the ER to arrange an advanced CT scan to identify these occult fractures in highly suspect cases.

Recent advances in artificial intelligence using deep learning techniques, such as deep convolutional neural networks (DCNNs), have shown remarkable results for a range of medical tasks as well as for human experts [10,11,12,13,14]. A growing number of studies support that deep learning networks can be trained to identify fractures in orthopedic radiographs with a satisfactory accuracy [15,16]. Although deep learning has been applied to fracture detection for radiological diagnoses, nondisplaced FNFs are often overlooked in a misdiagnosis, which may result in patients with nondisplaced fractures that deteriorate into displaced fractures. Therefore, we propose a new direction-aware fracture-detection network, termed DAFDNet, for the automatic detection of FNFs on anterior–posterior pelvic radiographs. It is well known that the Gabor filter is a differentiable band-pass filter with adjustable scales and orientations, and therefore, it has been integrated into DCNNs [17,18,19]. Garden type I and Garden type II FNFs present different orientations and frequencies in the frequency space, depending on the patient’s imaging location and conditions. By integrating a Gabor filter into the DCNN, the filter is able to fix the optimal parameters and help the DCNN learn robust feature presentations. We present this study to validate the accuracy of a DCNN in detecting nondisplaced FNFs, and it showed substantial improvements in performance. This study utilized a deep learning network to help physicians improve the diagnostic correctness compared to the current traditional manual orthopedic evaluations.

## 2. Materials and Methods

### 2.1. DCNN for FNFs

Recently, DCNN-based methods have shown a great potential efficiency in many areas of medical diagnoses and have encouraged further applied research [20]. The use of a DCNN can reduce the need for expensive computed tomography (CT) and magnetic resonance imaging (MRI) scans, and its automotive and accurate detection results can reduce the burden on clinicians for the urgent identification of fractures [12]. However, the feasibility and efficiency of detecting FNFs using a DCNN remain challenging and have not been fully investigated, especially for the occult representations of Garden I and II. To the best of our knowledge, typical DCNN-based methods [16,21,22,23,24], such as U-Net [21] and DenseNet [24], can be applied to detect FNFs. These types of fractures may disappear after a series of convolutional operations with the depth of the layers due to tiny variations in the grayscale distribution in these regions in the radiographic images.

### 2.2. Gabor Filter

A two-dimensional Gabor filter is a directional band-pass wavelet filter that is the multiplication of a Gaussian function and a cosine function, defined as follows [17]:

(1) $$G_{u,v}(z)=\frac{{\left\|k_{u,v}\right\|}^{2}}{{\left(2\pi \right)}^{2}}e^{-({\left\|k_{u,v}\right\|}^{2}{\left\|z\right\|}^{2}/2{\left(2\pi \right)}^{2})[e^{ik_{u,v}z}-e^{-{\left(2\pi \right)}^{2}/2}]}$$

where $k_{u,v}=k_{v}e^{ik_{u}}$, $k_{v}=(\pi /2)/{\sqrt{2}}^{\left(v-1\right)}$, $k_{u}=u\frac{\pi }{U}$, and *v* and *u* are the frequency and orientation, respectively. *U* stands for the total number of directions, and we set up 8 different angles to find the directionality of the feature. Substantially, the Gabor transform is a windowed short-time Fourier transform that is able to extract features locally or of certain frequency components. The direction and frequency selection properties make the Gabor filter sensitive to certain types of boundaries. In radiography, the orientations of nondisplaced FNFs are related to the patient’s position, where the frequency components lie in certain ranges. Therefore, in our study, a multiple-direction Gabor filter with adjusted frequency bands was engaged as an input layer to the DCNN to detect tiny changes in the grayscale distribution in Garden type I and type II fractures.

### 2.3. Attention Mechanism

The attention mechanism was invented to tell the DCNN where or what features to focus on, which was demonstrated to significantly improve the effectiveness of the model performance [25,26]. The squeeze-and-excitation attention network (SENet) exploits the squeeze function and the excitation function, i.e., the global average pooling operation and the sigmoid function, respectively, to encode inter-channel information [27]. This simple and innovative model provides a significant performance improvement for DCNNs, but ignores the location information that is important for capturing features. Therefore, several extension studies have proposed solutions such as the bottle attention module (BAM) [28], the convolutional block attention module (CBAM) [29], and spatial and channel-wise attention (SCA) [30] to further extract spatial and channel information and improve the network effectiveness. The self-attention DCNN model attention-in attention network (A^2^Net) divides the attention branches into attention and non-attention branches to maximize the use of high-contributing information and minimize the suppression of redundant information [31]. Although A^2^Net exhibits an excellent performance, the large amount of computation requires significant hardware facility costs. In summary, this study used the SCA strategy to obtain spatial and channel-wise information.

### 2.4. Direction-Aware Segmentation Network

In this section, we introduce the implementation details of the proposed DAFDNet model, including the attention mechanism ghost convolution and the details of the model architecture.

### 2.5. Squeeze-and-Excitation Ghost Convolution

**Figure 2:**
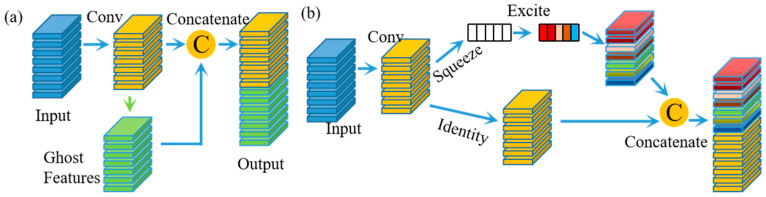
Original ghost convolution and SE ghost module. (**a**) Ghost convolution, which uses a simple linear transformation to generate more features; (**b**) SE ghost module, which incorporates the SE attention mechanism into the ghost module to discriminate the weight of each channel.

GhostNet was first proposed in reference [32] to reduce the computation consumption by replacing the ordinary convolution with a simple linear transformation. A ghost module divides the result of convolution into two parts. The first part involves ordinary convolution, while the other part uses a series of linear transformations to generate more feature maps, as shown in Figure 2a. By using this strategy, the lightweight ghost module produces more feature maps with inexpensive operations and performs better than other lightweight DCNNs, which also accelerates the learning process. However, the linear transformation does not focus on cross-channel relationships, which have proven to be robust in object detection. Therefore, each squeeze-and-excitation (SE) block consisting of global average pooling and two fully connected layers was embedded in the ghost module instead of the linear transformation, as shown in Figure 2b. The weights calculated from the SE blocks were then multiplied with the input convolution results by channel and concatenated with the original convolution results to generate the final feature maps.

### 2.6. Model Architecture

**Figure 3:**
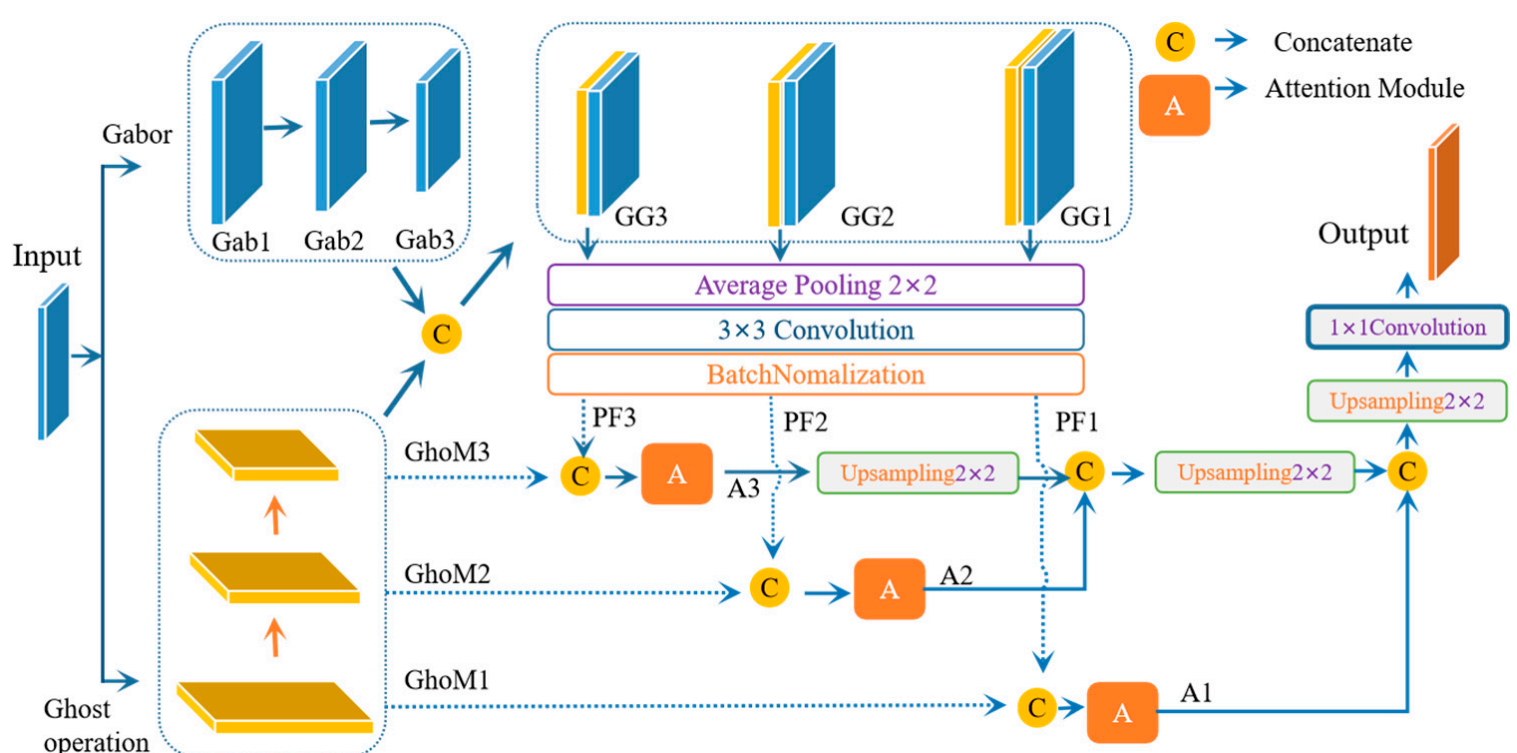
Schematic diagram of the proposed DAFDNet.

As shown in Figure 3, DAFDNet uses the popular encoder–decoder framework to first encode the input image by focusing on the attention ghost convolutional module and Gabor-filter convolution. The detailed structure of the DAFDNet framework is shown in Table 2. The input image is manipulated with the ghost module, and then two down-sampling operations are performed to gain global feature maps with different resolutions, i.e., *GhoM*_1_, *GhoM*_2_, and *GhoM*_3_. The output feature map *GhoM_i_* in the ghost module is represented as follows:

*GhoM*_*i*_ = *GhostConv*(*G*_*i*_, *L*_*i*_), *i* = 1, 2, … n (2)

where *L_i_* is the *i*th input feature map and *G_i_* is the *i*th ghost module.

In the process of bypassing the Gabor convolution, we used an 8-direction Gabor with diverse scales to extract the boundary of the FNF, and then performed two down-sampling operations to generate two additional batches of shrinking feature maps. Then, we obtained the feature maps *Gab_i_* from the Gabor convolution, which are represented as follows:

*Gab_i_* = *GaborConv*(*GF_i_*(*θ*,*s*), *L_i_*) *i* = 1, 2, … n (3)

where *GF_i_*(*θ,s*) is the Gabor filter with directions *θ* and scales *s.* Afterwards, in our study, *Gab*_1_, *Gab*_2_, and *Gab*_3_ were concatenated with *GhoM*_1_, *GhoM*_2_, and *GhoM*_3_, respectively, to obtain the aggregated feature maps *GG*_1_, *GG*_2_, and *GG*_3_, expressed by:

*GG*_*i*_ = *Concat*(*Gab_i_*, *GhoM_i_*) *i* = 1, 2, … n (4)

Then, the extracted features were refined with a 2 × 2 average pooling layer, a 3 × 3 convolution layer, and a batch normalization layer to obtain the *PF_i_* feature map. We concatenated the corresponding *GhoM_i_* and *PF_i_* with the same resolution to obtain *A_i_*, where the results were processed with the attention module, respectively. The feature maps with a lower resolution, such as *A*_3_ and *A*_2_, as shown in Figure 3, were resized with a 2 × 2 up-sampling layer before being concatenated to the feature maps with a higher resolution. In general, the process can be expressed as follows:

(5) $$\left\{\begin{array}{c} {Concat}_{i+1}=Concat({Upsampling(A}_{i+2}),A_{i+1})\\ {Concat}_{i}=Concat({Upsampling(A}_{i+1}),A_{i}) \end{array}i=1,2,...,n\right.$$

Hence, the output of our network was the result of the sequential operations of Equations (2)–(5) with a 2 × 2 up-sampling layer to recover the feature size as the input image, followed by a 1 × 1 convolution layer to reduce the dimension of the channels.

The loss function of DAFDNet is the mean-squared error, which can be expressed as:

(6) $$L(\vartheta )=\frac{1}{N}\sum_{i=1}^{N}{\left\|DASNet(I_{i}^{IN})-I_{i}^{GT}\right\|}_{2}$$

where ϑ represents the learnable parameter of DAFDNet and ${\left\|.\right\|}_{2}\;$is L_2_-norm. $I_{i}^{IN}$ and $I_{i}^{GT}$ denote the input images and the corresponding ground truth, respectively.

## 3. Experiments and Results

### 3.1. Dataset and Metrics

We extracted radiological images of the anterior–posterior view of the pelvis of 240 patients with nondisplaced ipsilateral FNFs (Garden type I and II) noted in relevant radiologists’ reports from the China Medical University Hospital (CMUH, Taichung, Taiwan) between 2018 and 2020, taken from the PACS (picture-archiving and communication system) database identified through the RIS (radiology information system). This study was approved by the institutional review board (IRB number: CMUH111-REC2-110). The inclusion criteria for patient selection were individuals who had been diagnosed with nondisplaced femoral neck fractures that were classified as Garden type I or II and patients with diagnostic reports. Individuals with displaced femoral neck fractures, preexisting implanted hardware around the fracture site, or musculoskeletal neoplasms were excluded. These 240 unilateral nondisplaced fractures were cut into 480 right and left joints, of which 240 were normal and 240 were nondisplaced fractures. There was no interaction with the patients directly, as we acquired de-identified data. This study was in accordance with the ethical standards of the institutional and national committee on human experimentation and conducted according to the guidelines of the Declaration of Helsinki. These radiographic images were rotated and rescaled with randomized rotations of the images from −15 to +15 degrees and a magnification reduction of 0.05 times to increase the number of frames to 3840, of which 3000 were used for training and 840 were used for testing. Two senior orthopedic surgeons were involved in the annotation, independently annotating the femoral neck part and the fracture line. In our algorithm, the femoral neck part was used to train the DCNN for ROI segmentation and the fracture line was used to train the DCNN for fracture detection. All labeled images were made under the guidance of a professional orthopedist, and new images of a 1024 × 1024 pixel size were extracted accordingly to reduce the computing time. We used the intersection-over-union (IOU) value between the FNF region and the labeled region as the assessing metrics, defined as *IOU =* (*A∩B*)*/*(*A*∪*B*), where *∩* and ∪ denote the intersection and union of two sets, *A* is the intersection of the predicted region and label region, and *B* is the predicted region. In addition to this, we used Dice and Jaccard as evaluation indicators with the following formulas:

(7) $$Dice=\frac{2\times |A\cap B|}{\left|A\right|+|B|}$$

(8) $$Jaccard=\frac{A\cap B}{A\cup B}$$

where *A* is the predicted region and *B* is the label region.

### 3.2. Implementation Details

**Figure 4:**
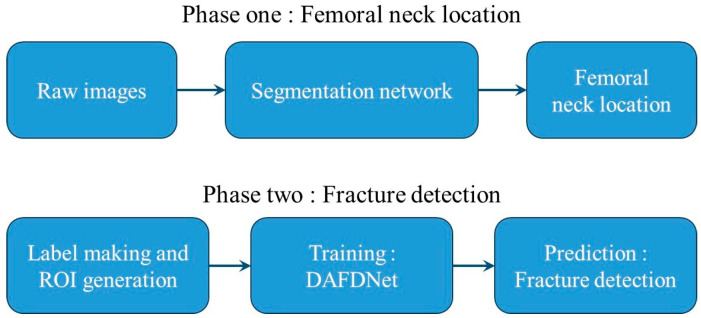
Workflow of the fracture-detection strategy. Two phases are included: femoral neck localization and fracture detection.

Figure 4 shows the FNF detection strategy used in this paper, which consisted of two phases, namely femoral neck localization and fracture detection. In the first stage, the original image was fed into a segmentation network with matching and alternative methods to accurately localize the femoral neck. In the second stage, a surgeon-made label-trained network was used to localize the exact location of the fracture after the output femoral neck image in the first stage.

We augmented the data by rotating and rescaling the images and labels with various degrees and scales. During the training process, 12 images were randomly chosen as the input in each training batch. The model was trained using the Adam optimizer with a learning rate initialized to 1 × 10^−5^ and set to 4 × 10^−5^ in steps of 1 × 10^−5^. Two typical DCNNs, namely U-Net [21] and DenseNet [24], were used as the comparison algorithms, and their codes were downloaded from GitHub, shared by the original authors. Up-sampling layers were added into DenseNet to achieve femoral neck segmentation. The corresponding parameters of these three methods were optimized until the network converged.

### 3.3. Results and Comparison

**Figure 5:**
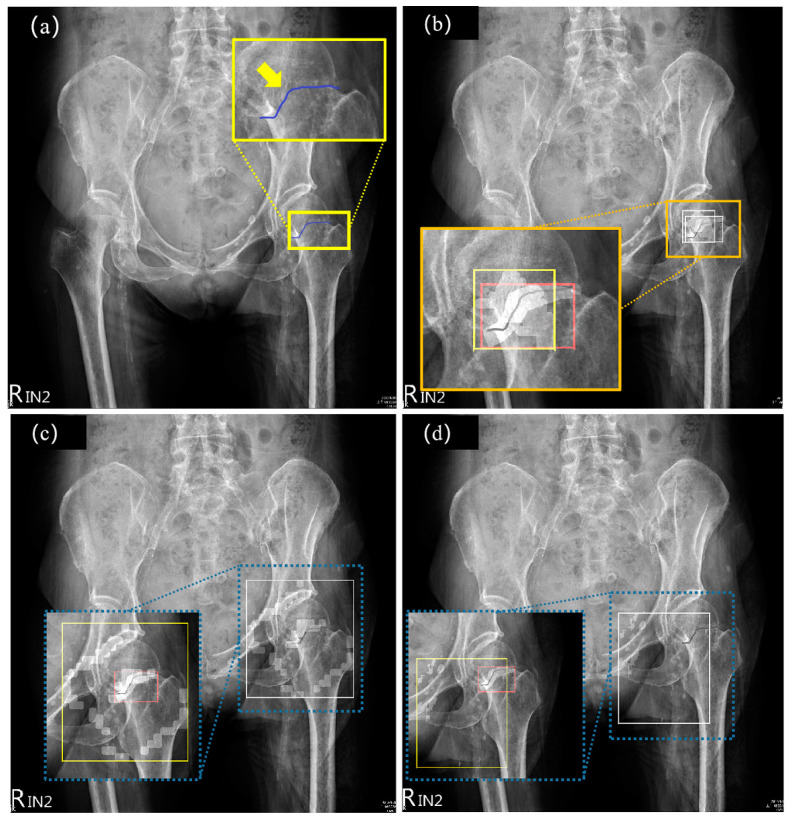
Radiographic images of pelvis and FNFs and the detection results of U-Net, DenseNet, and our proposed DAFDNet. The arrow indicates the location of the FNF and the dashed line indicates the zoomed-in area. (**a**) Imaging of the pelvis with FNF labeled by blue lines in the magnified view. (**b**) Fracture detected by DAFDNet, (**c**) fracture detected by U-Net, and (**d**) fracture location detected by DenseNet. As shown in the enlarged view, the fracture-detection range for each method is in the yellow rectangle and the physician-delineated labels are in the red rectangle.

Figure 5 shows the pelvic radiographic images with FNFs and the comparison of the detection results of U-Net, DenseNet, and our proposed DAFDNet. U-Net is more often used in image segmentation, and can roughly cut out the contour of the target, but cannot handle cracks without a displacement fracture well, because the cracks in the image are very small and inconspicuous, and U-Net is more suitable for dealing with more obvious and rough segmentation, but cannot deal with such small cracks. DenseNet is a related method used for image classification and detection; with the characteristic of dense connections, it can extract the features of smaller objects in the image, which strengthens the feature reuse and reduces the number of parameters, but in the process of feature extraction, the down-sampling method ignores the information of many small and dense objects, which reduces the accuracy of detection, and in the deeper layers of DenseNet, it may still face the problem of disappearing gradients [33], so it may not be suitable for dealing with small cracks. As shown in the enlarged view of Figure 5a, the fractures were labeled by an experienced surgeon and delineated with blue lines. The coordinates of both the labeled and predicted results are calculated and plotted in different colors in Figure 5b–d, where the labeled fracture region is plotted in red, while the predicted fracture region results are plotted in yellow. The detection results show that our proposed method was the closest to the actual size and area of the label region, while the other methods obtained results with an area several times larger than the labeled region. Therefore, our proposed method gained the largest IOU value among the three methods, which indicates that the fracture detected by our proposed method was the closest to the ground truth.

**Figure 6:**
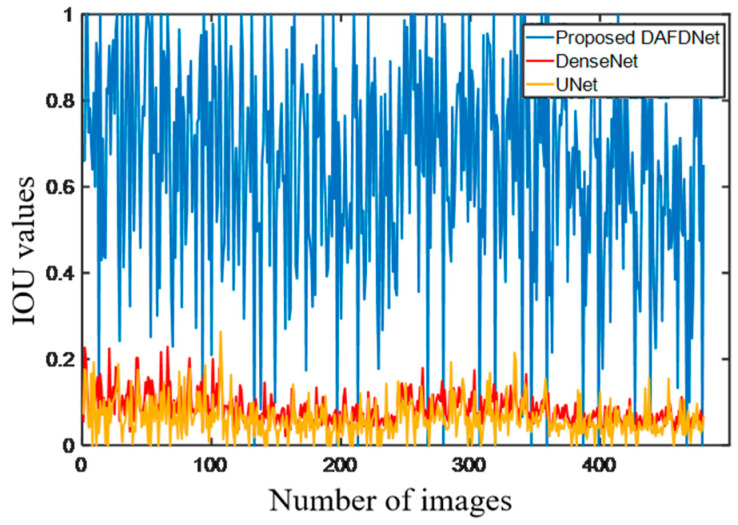
Comparison of IOU values of all the test images predicted by the three methods. Most of the IOU values of DAFDNet were larger than those of DenseNet and U-Net.

Figure 6 shows a comparison of the IOU values of our proposed DAFDNet, DenseNet, and U-Net. We can see that DAFDNet outperformed the other two methods, and most of the IOU values of DAFDNet were much larger than those of DenseNet and U-Net.

In Table 3, we divided the IOU values into three categories. A total of 73.1% of the DAFDNet results were above 0.5 (or 50%), while none of the other two methods achieved this. A total of 21.7% of the DAFDNet results were between 0.2 and 0.5 (or [20%, 50%]), while more than 90% of the DenseNet and U-Net comparison method results were close to 0.1 (or [0%, 20%]). The table also calculates the average IOU values, which were 0.648, 0.084, and 0.062 (or 64.8%, 8.4%, and 6.2%) for DAFDNet, DenseNet, and U-Net, respectively. The average Dice values were 0.542, 0.06, and 0.041 (or 54.2%, 6.0%, and 4.1%) for DAFDNet, DenseNet, and U-Net, respectively. The average Jaccard values were 0.426, 0.031, and 0.021 (or 42.6%, 3.1%, and 2.1%) for DAFDNet, DenseNet, and U-Net, respectively. The simulation results showed that DAFDNet outperformed the U-Net and DenseNet methods in terms of the IOU value, Dice value, and Jaccard value.

**Figure 7:**
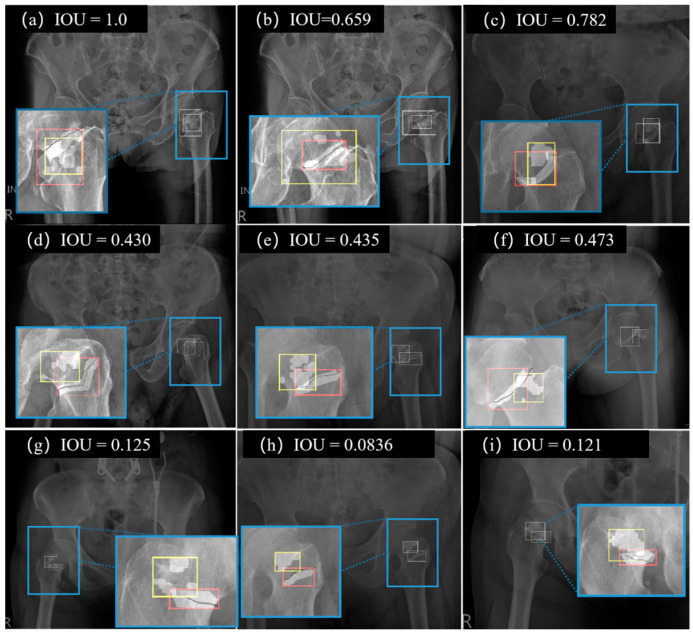
Detection results of DAFDNet with different IOU values. In the enlarged views, the red rectangle illustrates the region of ground truth and the yellow rectangle is the fracture region predicted by DAFDNet.

Figure 7 shows the detection results of DAFDNet for different IOU values. The IOU values are divided into three classes, where values above 50% are shown in a–c, those between 20% and 50% are shown in d–f, and values below 20% are shown in g–i. In the enlarged view of all sub-images, the red rectangle indicates the labeled region (ground truth) and the yellow rectangle is the fracture region predicted by DAFDNet. It was concluded that DAFDNet achieved a better performance than the DenseNet and U-Net comparison methods. The diagnostic correctness of DAFDNet exceeded 94.8% and the DAFDNet method could assist general practitioners and orthopedic surgeons in the initial diagnosis of Garden type I and II fractures to avoid misclassification and improve the diagnostic correctness.

## 4. Discussion and Conclusions

In this study, we proposed a new method for detecting FNFs. The results show that our method was effective in detecting precise fracture locations and outperformed other comparative methods. Our proposed method is implemented in the localization and detection phases, i.e., localization of the femoral neck and fracture detection. The benefit of the localization phase is that, by localizing the ROI from the original image, the input data size of the DCNN can be greatly reduced. On the one hand, the computation time is saved to a great extent, and on the other hand, the disturbances, such as regions with a similar gray distribution in the pelvis image, can be excluded to improve the accuracy of detection. In the fracture-detection stage, because the orientation of a fracture is random, DAFDNet introduces an orientation-aware algorithm to detect fracture directionality. The use of a band-pass Gabor filter enabled the network to detect image gray changes by adjusting its frequency and orientation. In addition, attention mechanism and ghost convolution were also involved to improve the performance of DAFDNet.

Although deep learning has made great advances in medical image processing, few publications have shown a clinical utility for detecting FNFs, especially for nondisplaced Garden I and Garden II fracture detection. The success of our study in detecting the precise location of nondisplaced fractures provides the first evidence that DCNNs can help physicians improve the diagnostic accuracy of nondisplaced Garden I and Garden II fractures. As shown by the predicted rectangular and IOU values of the fractures, our proposed method obtained better results than a physician diagnosis. However, fractures were not detected in more than 5.2% of the images tested, due to the poor contrast of the images. Therefore, a better radiographic image quality would greatly improve our approach.

Elderly patients suffering from nondisplaced FNFs may have been misrecognized negatively in plain films in the first place. The overall sensitivity to hip fractures in plain film radiography (anteroposterior pelvis and lateral hip view) is about 90–98% [34]. A surgical intervention for nondisplaced FNFs usually involves a closed reduction and an internal fixation with multiple cannulated screws or sliding hip screws. Early surgery (within 48 h of admission) after a hip fracture reduces the hospital stay and may also reduce complications and mortality [35]. Delayed recognized or misdiagnosed nondisplaced FNFs may lead to the further displacement of the fracture site. Displaced FNF is the major risk factor of avascular necrosis of the femoral head and the nonunion of fractures. Elderly patients with displaced FNFs should be treated with bipolar hemiarthroplasty. Compared with a closed reduction and internal fixation with multiple cannulated screws, the surgical time of bipolar hemiarthroplasty is significantly longer, and perioperative blood loss is significantly increased [36]. Therefore, recognizing nondisplaced FNFs as soon as possible is crucial for better outcomes.

## Figures and Tables

**Table 1:** Comparison of misclassification rate in nondisplaced FNFs in radiographs among different professional physicians.

Profession	Misrecognized Fracture Rate	*p*-Value ^1^
ER doctor	4.19%	<0.0001
PGY-1 doctor	7.87%	<0.0001
Senior orthopedic doctor	2.44%	<0.0001

^1^ The *p*-values were estimated using the chi-squared test.

**Table 2:** The detailed structure of the DAFDNet framework.

Stage	Layer	Size	Channel
Input	Input	1024 × 1024	1
Gabor	*Gab1*	1024 × 1024	32
	*Gab2*	512 × 512	32
	*Gab3*	256 × 256	32
Ghost	*GhoM1*	1024 × 1024	32
	*GhoM2*	512 × 512	64
	*GhoM3*	256 × 256	128
Gabor + Ghost	*GG1*	1024 × 1024	64
	*GG2*	512 × 512	96
	*GG3*	256 × 256	150
PF	PF1	1024 × 1024	32
	PF2	512 × 512	64
	PF3	256 × 256	128
Attention Module	*A1*	1024 × 1024	64
	*A2*	512 × 512	128
	*A3*	256 × 256	256
Output	Output	1024 × 1024	1

**Table 3:** Statistical results of the IOU, Dice, and Jaccard values.

	Methods	U-Net [21] (%)	DenseNet [24] (%)	DAFDNet (%)
IOU (%)				
[50, 100]		0	0	73.1
[20, 50]		0.4	1.6	21.7
[0, 20]		99.6	98.4	5.2
Average IOU		6.2	8.4	64.8
Dice		4.1	6.0	54.2
Jaccard		2.1	3.1	42.6

## Data Availability

The datasets used or analyzed during the current study are available from the corresponding author upon reasonable request.
